# Supplementary figures and images for: Serum susceptibility of Escherichia coli and its association with patient clinical outcomes
Source: PLoS One. 2024 Jul 29;19(7):e0307968. doi: 10.1371/journal.pone.0307968 (PMC11285940; doi:10.1371/journal.pone.0307968)

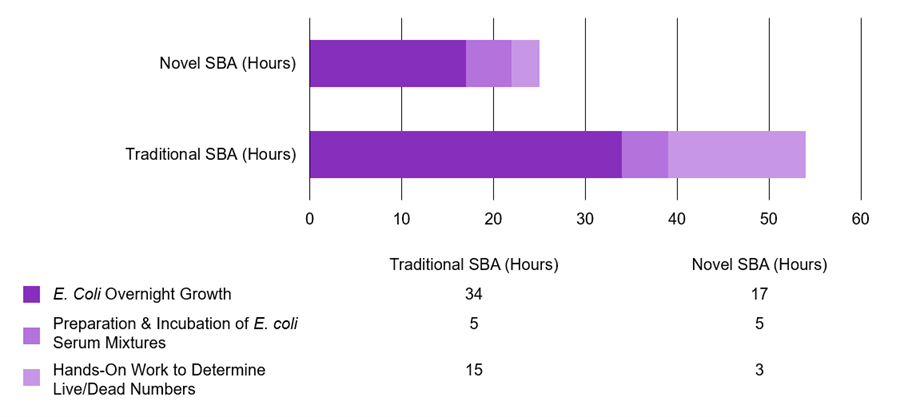

Supplement: S1 Fig — The estimates are time required for a single person to perform the experiment with 16 E. coli strains (0%, 25%, and 50% serum) in duplicate. For the flow cytometry-based SBA, this corresponds to one full 96-well plate. (TIF) [file pone.0307968.s001.tif]

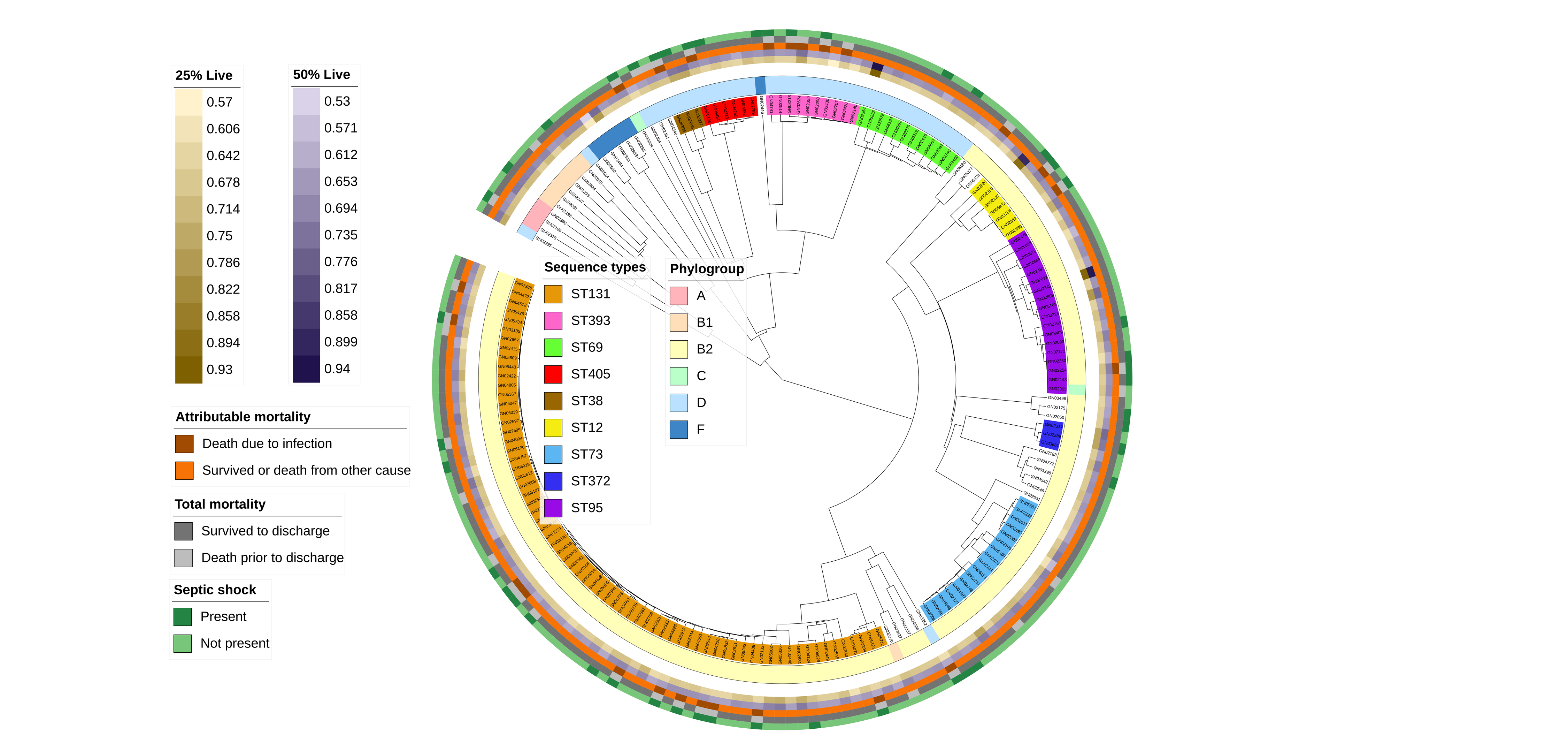

Supplement: S2 Fig — The innermost two colored range indicate the most common multilocus sequence types and phylogroup, respectively. The outer colored strips indicate the live bacteria proportions associated with exposure to 25% serum (yellow) and 50% serum (purple) as well as the corresponding patient clinical data of attributable in-hospital mortality (orange), total in-hospital mortality (gray), and septic shock (green). (TIF) [file pone.0307968.s002.tif]
