# Supplementary material for: Serum susceptibility of Escherichia coli and its association with patient clinical outcomes
Source: PLoS One. 2024 Jul 29;19(7):e0307968. doi: 10.1371/journal.pone.0307968 (PMC11285940; doi:10.1371/journal.pone.0307968)
Supplement: S1 Table — Covariates with p<0.15 in univariable logistic regression analyses were included in a multivariable logistic regression model. The final multivariable logistic regression model is shown here. P-values ≤0.05 are in bold. (DOCX) [file pone.0307968.s003.docx]

| **Covariate** | **Odds Ratio** | **95% CI** | **p-value** |
| --- | --- | --- | --- |
| **Age** | 1.04 | 1.01 – 1.08 | **0.02** |
| **Route of BSI**^1^ |  |  |  |
| Community acquired/Healthcare-associated | 0.36 | 0.13 – 0.99 | **0.05** |
| Community-acquired/Non-healthcare-associated | 0.44 | 0.10 – 1.69 | 0.24 |
| **Hemodialysis** | 5.43 | 1.61 – 18.92 | **<0.01** |
| **Source of BSI**^2^ |  |  |  |
| Abscess | 3.64 | 0.42 – 24.79 | 0.20 |
| Biliary Tract | 0.60 | 0.03 – 3.91 | 0.65 |
| Line | 1.99 | 0.09 – 20.46 | 0.65 |
| Other | 3.43 | 0.98 – 11.84 | **0.05** |
| Pneumonia | 4.45 | 0.80 – 25.31 | 0.08 |
| Skin/soft tissue infection | 1.35 | 0.07 – 9.53 | 0.80 |
| Unknown | 4.65 | 1.53 – 14.83 | **<0.01** |
| **Chronic health APACHE II score** | 1.07 | 0.86 – 1.38 | 0.55 |
| ^1^ Reference is hospital-acquired infection  ^2^ Reference is urinary tract source | | | |
