# Supplementary material for: Serum susceptibility of Escherichia coli and its association with patient clinical outcomes
Source: PLoS One. 2024 Jul 29;19(7):e0307968. doi: 10.1371/journal.pone.0307968 (PMC11285940; doi:10.1371/journal.pone.0307968)
Supplement: S2 Table — Covariates with p<0.15 in univariable logistic regression analyses were included in a multivariable logistic regression model. The final multivariable logistic regression model is shown here. P-values ≤0.05 are in bold. (DOCX) [file pone.0307968.s004.docx]

| **Covariate** | **Odds Ratio** | **95% CI** | **P-value** |
| --- | --- | --- | --- |
| **Age** | 1.03 | 1.00 - 1.08 | 0.08 |
| **Serum Susceptibility** | < 0.01 | < 0.01 - 86.19 | 0.26 |
| **Hemodialysis** | 3.62 | 0.82 - 15.43 | 0.08 |
| **Corticosteroid** | 0.13 | < 0.01 - 0.73 | 0.06 |
| **Source of BSI**^1^ |  |  |  |
| Abscess | 6.25 | 0.87 – 15.43 | **0.05** |
| Biliary Tract | 0.89 | 0.04 – 6.48 | 0.92 |
| Line | < 0.01 | NA | 0.99 |
| Other | 5.64 | 1.39 – 23.95 | **0.02** |
| Pneumonia | 2.77 | 1.37 – 23.11 | 0.40 |
| SSTI | 3.76 | 0.93 – 15.44 | 0.06 |
| Unknown | 0.13 | < 0.01 – 0.73 | 0.06 |
| ^1^ Reference is urinary tract source | | | |
