# Supplementary material for: Serum susceptibility of Escherichia coli and its association with patient clinical outcomes
Source: PLoS One. 2024 Jul 29;19(7):e0307968. doi: 10.1371/journal.pone.0307968 (PMC11285940; doi:10.1371/journal.pone.0307968)
Supplement: Attachment — Submitted filename: Recommendations.pdf [file pone.0307968.s005.pdf]

Recommendations: Serum Susceptibility of *Escherichia coli* and its Association with Patient Clinical Outcomes. Manuscript number: PONE-D-24-09625

Reviewer: Naoise McGarry, PhD

Recommendations:

Line 43/44 – 'The complement system is a regulated network of proteins in the blood that is composed of three pathways' to indicate that the three pathways are distinct, e.g. 'The complement system is a regulated network of proteins in the blood with three potential pathways for activation'.

Line 48 – provide purpose of MAC complex to indicate that MAC insertion causes cell lysis

Line 103 – Remove line 'This is the standard approach for performing an SBA' and change to 'As in the standard approach for performing an SBA, E. coli isolates were streaked onto … ' to continue into next sentence.

Line 180 – Consider performing bioinformatic analysis to determine bacterial phylogroup via Clermont typing or EzClermont (https://ezclermont.hutton.ac.uk/)- can run off PC or command line. The results would likely be interesting as you might find the isolates more resistant to serum belong to the B2/D phylogroups and those which are sensitive may belong to another phylogroup, indicating the infection was driven by immune status of the host, rather than virulence.

Line 210 – Perform statistical analysis of results from your SBA vs Flow methods to strengthen the argument that results are reproducible and comparable to the traditionally used method. ANOVA would be a good test.

Line 250 – you mention 'bacterial characteristics' in table, in reference to antibiotic resistance yet there is no indication in methods for how this was determined. Are you referring to phenotypic or genotypic resistance? If phenotypic, were AMR genes identified in the whole genome sequencing analysis? If the AMR experiments were performed on the isolates in the previous paper your group has published, ensure to reference that in the text as well as in methods e.g. 123 isolates were found to be MDR, as detailed in (ref) OR 'as determined by the presence of genes or mutations associated with resistance'.

Line 269 – If possible, include sequence types on legend in supplementary figure regarding bacterial genotype. If phylogroup is determined as suggested above, include also.

Line 314 – Another study has explored the serum resistance and general characteristics of 20 *E. coli* bloodstream isolates associated with patient mortality in an Irish hospital - PMID: 26518234. The aforementioned study showed that in the *E. coli* isolates which displayed *in vitro* serum sensitivity, the patients had several co-morbidities often associated with poor outcomes. I would suggest looking for a similar trend among your isolates, to highlight that both bacterial (virulence) and host factors (health status) can play into morbidity and patient outcome. Similarly, I would include that study in your reference list.

Line 331 – I suggest correlating serum sensitive isolates to patient data as mentioned above. If not possible due to ethics, state that such a comparison would prove useful but was not possible due to ethics and consider referencing papers which did so.
